# Plantar plate pathology is associated with erosive disease in the painful forefoot of patients with rheumatoid arthritis

**DOI:** 10.1186/s12891-017-1668-0

**Published:** 2017-07-18

**Authors:** Heidi J Siddle, Richard J Hodgson, Elizabeth M A Hensor, Andrew J Grainger, Anthony C Redmond, Richard J Wakefield, Philip S Helliwell

**Affiliations:** 1 Leeds Institute of Rheumatic and Musculoskeletal Medicine, University of Leeds, Chapel Allerton Hospital, Chapeltown Road, Leeds, LS7 4SA UK; 2 NIHR Leeds Biomedical Research Centre, Leeds Teaching Hospitals NHS Trust, Chapel Allerton Hospital, Chapeltown Road, Leeds, LS7 4SA UK; 3 Centre for Imaging Sciences, University of Manchester, Oxford Road, Manchester, M13 9PL UK; 4 Department of Radiology, Leeds Teaching Hospitals NHS Trust, Chapel Allerton Hospital, Chapeltown Road, Leeds, LS7 4SA UK

**Keywords:** Rheumatoid arthritis, Foot, Metatarsophalangeal joint, Erosion, Magnetic resonance imaging, Plantar plate

## Abstract

**Background:**

Disease-related foot pathology is recognised to have a significant impact on mobility and functional capacity in the majority of patients with rheumatoid arthritis (RA). The forefoot is widely affected and the metatarsophalangeal (MTP) joints are the most common site of symptoms. The plantar plates are the fibrocartilaginous distal attachments of the plantar fascia inserting into the five proximal phalanges. Together with the transverse metatarsal ligament they prevent splaying of the forefoot and subluxation of the MTP joints. Damage to the plantar plates is a plausible mechanism therefore, through which the forefoot presentation, commonly described as ‘walking on pebbles’, may develop in patients with RA.

The aims of this study were to investigate the relationship between plantar plate pathology and clinical, biomechanical and plain radiography findings in the painful forefoot of patients with RA. Secondly, to compare plantar plate pathology at the symptomatic lesser (2nd-5th) MTP joints in patients with RA, with a group of healthy age and gender matched control subjects without foot pain.

**Methods:**

In 41 patients with RA and ten control subjects the forefoot was imaged using 3T MRI. Intermediate weighted fat-suppressed sagittal and short axis sequences were acquired through the lesser MTP joints. Images were read prospectively by two radiologists and consensus reached. Plantar plate pathology in patients with RA was compared with control subjects. Multivariable multilevel modelling was used to assess the association between plantar plate pathology and the clinical, biomechanical and plain radiography findings.

**Results:**

There were significant differences between control subjects and patients with RA in the presence of plantar plate pathology at the lesser MTP joints. No substantive or statistically significant associations were found between plantar plate pathology and clinical and biomechanical findings. The presence of plantar plate pathology was independently associated with an increase in the odds of erosion (OR = 52.50 [8.38–326.97], *p* < 0.001).

**Conclusion:**

The distribution of plantar plate pathology at the lesser MTP joints in healthy control subjects differs to that seen in patients with RA who have the consequence of inflammatory disease in the forefoot. Longitudinal follow-up is required to determine the mechanism and presentation of plantar plate pathology in the painful forefoot of patients with RA.

**Electronic supplementary material:**

The online version of this article (doi:10.1186/s12891-017-1668-0) contains supplementary material, which is available to authorized users.

## Background

Forefoot pain and deformity is a common feature of rheumatoid arthritis (RA) [1]. Despite improvements in systemic disease management and local treatments the mechanism of metatarsophalangeal (MTP) joint disease is not fully understood [2–5].

Cadaveric studies of the forefoot in patients with RA have suggested that the characteristic forefoot deformities may result from a failure of the multi-segmental ligamentous system of the MTP joints and the dynamic effect of displacement of the plantar plates [6]. The plantar plates are the fibrocartilaginous distal attachments of the deeper layer of the plantar fascia, inserting into the five proximal phalanges. Together with the transverse metatarsal ligament they prevent splaying of the forefoot and subluxation of the MTP joints.

Inflammation of the synovium in RA can cause distension and stretching of the joint capsule which subsequently leads to instability. Together with repeated hyperextension of the MTP joint during gait, it is hypothesised that in patients with RA this may predispose the plantar plates to attenuation or rupture [7]. As a consequence, degeneration or loss of function of the plantar plate (plantar plate pathology) is a potential mechanism which may contribute to the characteristic forefoot deformity, often described as ‘walking on pebbles,’ may occur in patients with RA.

Recent exploratory studies using magnetic resonance imaging (MRI) have demonstrated that plantar plate pathology is common at the lesser (2nd-5th) MTP joints in patients with RA (79.2% of patients) and appears to be associated with features of disease and deformity at the lesser MTP joints [8, 9]. Plantar plate pathology was reported to be more common at the 4th and 5th MTP joints in patients with RA [8], in contrast to the predilection for the 2nd MTP reported previously in people without RA [10, 11]. This is supported by the frequency of plantar plate pathology seen at the 5th MTP joint, known to be the most common site of erosive change in the forefoot [12–14].

This study investigated the hypothesis that plantar plate pathology in RA is associated with specific features of disease at the lesser MTP joints; radiographic damage, deformity, higher peak plantar pressure, plantar callus formation and disease duration. The primary aim of this study was to use high resolution 3 Tesla (3T) MRI to investigate the relationship between plantar plate pathology and the clinical, biomechanical and plain radiography findings in the painful forefoot of patients with RA. The study also aimed to compare MRI reported plantar plate pathology at the symptomatic lesser MTP joints in patients with RA, with a group of healthy age and gender matched control subjects without forefoot pain.

## Methods

### Recruitment of participants

Local Ethical Committee approval [Leeds (West) Research Ethics Committee REC reference: 08/H1307/29] was received and informed written consent was obtained from all participants. Consecutive patients presenting to a specialist Rheumatology foot clinic with a diagnosis of RA and pain on the plantar aspect of their lesser MTP joints were invited to take part in the study. All patients were diagnosed according to the 1987 American College of Rheumatology revised criteria for RA [15], and were diagnosed prior to the introduction of the 2010 RA classification criteria [16]. The anatomy and pattern of pathological changes at the first MTP joint differs to that seen at the lesser MTP joints, therefore the first MTP joint was excluded. Control subjects were eligible to take part if they did not have RA or any other rheumatic disease, or any current or previous foot pain. Participants were excluded if they had a diagnosis of diabetes mellitus, peripheral vascular disease of the lower extremities, neurological disease with lower limb symptoms, a history of forefoot surgery or contraindications to having a MRI scan.

### Clinical, gait and x-ray measures

Demographic data, current medication and medical history were recorded for all participants.

Measurement of functional impairment and foot deformity were recorded for healthy control subjects and patients with RA. Current pain across the plantar MTP joint area was recorded using a 100 mm visual analogue scale (VAS) score, with the anchors “no pain” and “worst pain imaginable” [17]. All participants completed the Foot Impact Scale (FIS), a self-completed foot health outcome tool for RA [18]. Platto’s Structural Index was used to quantify the degree of forefoot structural deformity [19]. The presence of plantar callus and clinically reported subluxation at each lesser MTP joint was recorded by an experienced clinician (HJS).

Peak plantar pressure (kPa) at each lesser MTP joint was measured using an emed-SF pressure platform (Novel GmbH, Munich Germany) [20]. Using the second step method, the average of three barefoot measurements was recorded for each patient. A patient specific mask was applied to each lesser MTP joint to calculate the peak plantar pressure. In patients with RA only, current disease activity was quantified using a disease activity score (DAS 44) which included the MTP joints [15]. Standard dorsi-plantar radiographs were taken to identify the radiographic damage at each lesser MTP joint. Radiographs were read and scored by an experienced consultant rheumatologist (PSH) using the Larsen method, a 6-point grading system (0–5) for progression of radiographic joint damage [16].

### MRI protocol

All MRI sequences were acquired using a 3T Verio scanner (Siemens Healthcare, Erlangen, Germany) with an eight channel radio frequency knee coil. In patients with RA, the more symptomatic forefoot (determined by current VAS scores) was imaged and for control subjects the dominant forefoot was chosen. Participants lay in a supine position with their knee flexed and the forefoot was placed within the coil.

Intermediate-weighted, fat-suppressed sagittal (turbo spin echo (TSE), field of view (FOV) =130, acquisition time (TA) =6mins, repetition time (TR) =4500-5480 ms, echo time (TE) =34 ms, 1.5 × 0.5 × 0.3 mm resolution) and short axis (TSE, FOV = 130, TA = 7mins, TR = 3500-3760 ms, TE = 31-36 ms, 1.1 × 0.5 × 0.3 mm resolution) sequences, which had been previously optimised to demonstrate the plantar plate and joint [8], were acquired through the lesser MTP joints.

**Fig. 1:**
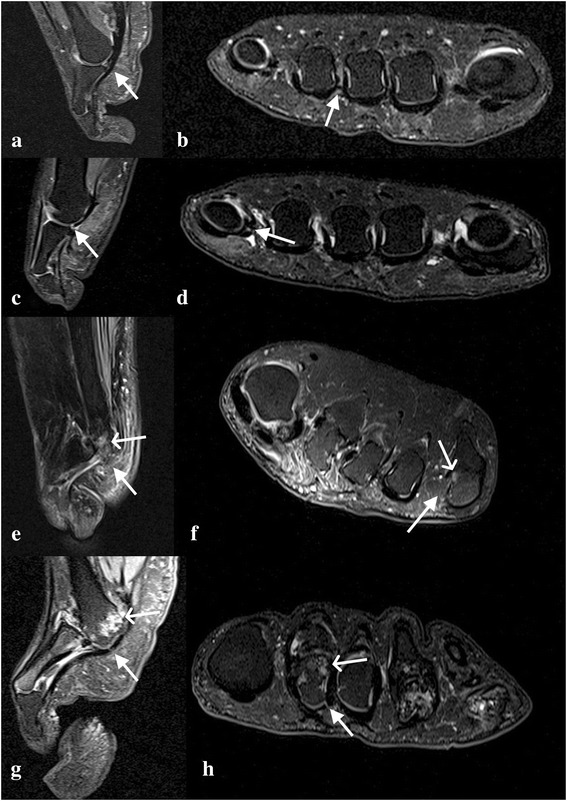
Intermediate-weighted, fat-suppressed sagittal (**a**) and short axis (**b**) MR images of 3rd MTP joint demonstrating an intact capsule and plantar plate (white arrow) in a control subject. Sagittal (**c**) and short axis (**d**) images of a central-distal plantar plate tear (white arrow) at the 5th MTP joint in a control subject. Sagittal (**e**) and short axis (**f**) images demonstrating an absent plantar plate (closed arrow) and erosion (open arrow) at the 5th MTP joint in a patient with RA. Sagittal (**g**) and short axis (**h**) images demonstrating a tear (high signal) (closed arrow) in the plantar plate and erosion (open arrow) of the 2nd MTP joint in a patient with RA

All MR images were read prospectively by two experienced musculoskeletal radiologists (RJH and AJG) who were blind to the participant group, and consensus on scoring was reached. Plantar plate pathology was defined as absence of (failure to visualise) the plantar plate (Fig. 1 e and f; closed arrow), or a full width or partial width tear (distal or proximal, medial, central or lateral) (Fig. 1 c and d; central-distal tear), including failure to visualise the medial or lateral distal insertions. In addition, the presence of central high signal intensity at the distal insertion was also recorded; a tear, rupture or discontinuity of the plantar plate has been described as an increase in signal or hyperintense focus at the insertion [7, 11] (Fig. 1 g and h; closed arrow).

### Statistical analysis

Analyses were performed with SPSS version 19.0.0.2 and Stata 12.1.

Control subjects were compared to patients with RA using Student’s independent sample t-tests for normally distributed interval data, Mann-Whitney U tests for ordinal data, and Pearson’s chi-square tests for categorical data.

In order to identify associations between features of disease and plantar plate pathology at the lesser MTP joints in patients with RA, initially a bivariable analysis was undertaken at the patient level using a Spearman’s rank correlation. Variables at the joint level were summarised as either the number of affected joints (plantar plate pathology, plantar callus formation, MTP joint subluxation) or the maximum score recorded (Larsen score, peak plantar pressure). Spearman’s rho >0.3 was chosen as the threshold for preliminary evidence of a substantive association, irrespective of statistical significance in this study.

High signal at the plantar plate distal insertion in patients with RA was compared with the reported presence of a tear for sensitivity, specificity, positive predictive value, negative predictive value and accuracy.

Associations between the measures of pathology recorded at each joint were investigated using multilevel regression modelling to address the clustering of joints (level 1) within patients (level 2). Likelihood ratio tests were conducted to show whether there was a significant degree of clustering of joints within patients, linear regression was used to model peak pressure recorded in each of the lesser MTP joints and the natural logarithm of the values was used to address non-normality of the residuals. Consequently, the regression coefficients represent percentage change in peak plantar pressure. Binary logistic regression was used to model the odds of the presence of i) plantar plate pathology, ii) presence of erosions (Larsen grade > 1), iii) MTP joint subluxation and iv) plantar callus formation. Bivariable models were supplemented on an exploratory basis with multivariable models to investigate whether plantar plate pathology was independently associated with the presence of erosions, MTP joint subluxation or plantar callus formation when controlling for contextual demographic and clinical variables.

In all modelling *p* < 0.1 was considered indicative of an association of potential interest warranting further investigation.

## Results

### Clinical, biomechanical and x-ray measures

Forty five patients with RA and 13 control subjects were approached to take part in the study; 41 patients with RA and ten control subjects met the inclusion criteria and took part in the study. Demographic characteristics and pain, and measures of functional impairment and foot deformity by group and comparisons between the control subjects and patients with RA are given in Table 1.

**Table 1:** Comparison of date between control subjects (*n* = 10) and patients with RA (*n* = 41)

Demographic characteristics and pain		Controls (*n* = 10)	RA (*n* = 41)	Test, statistic, significance
Age, yrs.:	mean (SD), range	55.6 (8.0), 42–72	55.3 (11.5), 25–77	Student’s t-test, *t* = 0.07, *p* = 0.283
Sex:	% female (n)	70.0% (7/10)	78.0% (32/41)	Chi-sq., χ^2^ = 0.29, *p* = 0.591
VAS pain, mm:	median (IQR), range	0.0 (0.0–0.3), 0–2	36.0 (18.0–70.0), 8–95	Mann-Whitney U, z = 4.87, *p* < 0.001
Functional impairment and foot deformity		Controls (*n* = 10)	RA (*n* = 41)	
FIS_IF_ score:	median (IQR)	1 (0–3)	13 (11–15)	Mann-Whitney U, z = 4.82, *p* < 0.001
FIS_AP_ score:	median (IQR)	0 (0–0)	16 (11–23)	Mann-Whitney U, z = 4.89, *p* < 0.001
Platto forefoot index:	median (IQR)	2 (1–2)	9 (5–10)	Mann-Whitney U, z = 3.71, *p* < 0.001
Gait velocity, cm/s:	mean (95% CI)	117.78 (4.67)	92.70 (4.38)	Student’s t-test, *t* = 3.92, *p* = 0.001
Subluxation:	% patients (n)	0.0% (0/10)	70.7% (29/41)	Chi-sq., χ^2^ = 16.40, *p* < 0.001
	% joints (n)	0.0 (0/40)	61.6% (101/164)	ML log reg^a^
Callus:	% patients (n)	60.0% (6/10)	43.9% (18/41)	Chi-sq., χ^2^ = 0.84, *p* = 0.360
	% joints (n)	17.5% (7/40)	17.7% (29/164)	ML log reg, z = −0.08, *p* = 0.939
Plantar plate pathology:	% patients (n)	20.0% (2/10)	85.4% (35/41)	Chi-sq., χ^2^ = 17.24, *p* < 0.001
	% joints (n)	5.0% (2/40)	46.6% (76/163)	ML log reg; z = 3.57, *p* < 0.001
Peak pressure, kPa: Peak pressure, kPa:	max per patient median (IQR) individual joint geometric mean	563.3 (454.6–773.8) 323.75	746.7 (555.0–1145.0) 399.41	Mann-Whitney U, z = 1.90, *p* = 0.058 ML lin reg; z = −1.66, *p* = 0.096

*AP* Activity and Participation, *Chi-sq.* Pearson’s chi-square, *FIS* Foot Impact Scale, *IF* Impairment and Footwear, *lin* linear, *log* logistic, *ML* multilevel, *reg* regression, *t* Student’s independent samples t-statistic, *VAS* Visual Analogue Scale, *z* normal distribution standardised statistic

^a^The multilevel model could not be run due to the fact that none of the control patients had any joints showing subluxation

In patients with RA the median (interquartile range [IQR]) disease duration was 6.0 (1.8–13.0) years, 31 (77.55%) patients were rheumatoid factor positive and the mean (standard deviation) DAS44 (CRP) was 2.80 (0.72). Thirty five (85.4%) patients with RA were taking a disease modifying anti rheumatic drug (DMARD), 16 patients (39%) were taking a biologic therapy, four patients (10%) were taking oral steroids and 20 patients (50%) were taking non-steroidal anti-inflammatory drugs (NSAIDs). Lesser MTP joint Larsen median (IQR) scores in patients with RA were 1.0 (0.5–2.0), 13/41 (31.7%) patients and 58/164 (35.4%) joints had a Larsen score of more than one.

### Plantar plate pathology on MRI

Plantar plate pathology is described initially at a patient level and all subsequent descriptions are described per lesser MTP joint.

At the patient level, in the control group all the plantar plates were present (Fig. 1 a and b; intact plantar plate at 3rd MTP joint). In the RA group one or more lesser plantar plates were absent in 15 (37%) patients. All the lesser plantar plates were absent in one patient. In the control group a tear was identified in only two (20%) patients. Tears in one or more of the lesser plantar plates were identified in 29 (71%) patients with RA.

At the joint level, in the control group tears were observed in two (5%) lesser plantar plates, both at 5th MTP joint (Fig. 1 c and d). In the RA group (data was available for 163 plantar plates; MR images were not suitable for scoring the plantar plate of the 5th MTP joint in one patient), 32/163 (20%) plantar plates were absent; seven were absent at 2nd MTP joints, six at 3rd MTP joints, seven at 4th MTP joints and 12 at 5th MTP joints. Tears were observed in 44 (34%) of the 131 remaining plantar plates; seven tears were reported in the plantar plates of the 2nd MTP joints, nine at 3rd MTP joints, 11 at 4th MTP joints and 17 at 5th MTP joints.

The 5th MTP joint was the most common site for plantar plate pathology in patients with RA (odds ratio [95% confidence interval] relative to 2nd MTP joint 9.20 [2.77–30.51], *p* < 0.001) and there was little difference between the 2nd, 3rd (1.16 [0.40–3.33], *p* = 0.788) and 4th (1.76 [0.61–5.03], *p* = 0.293) MTP joints.

Patient and joint level data for the presence of plantar plates, presence of tears, location of tears (width and length) and plantar plate pathology for control subjects and patients with RA is summarised in Table 2.

**Table 2:** Comparison of plantar plate pathology between control subjects (*n* = 10) and patients with RA (*n* = 41)

Pathology at the MTP joint level	Controls (*n* = 10) Joints (*n* = 40)	Patients (*n* = 41) Joints (*n* = 163^a^)
PP present:		
% patients with all 4 (n)	100.0 (10/10)	82.9% (34/41)
% joints (n)	100.0% (40/40)	80.4% (131/163)
Tear present:		
% patients (n)	20.0% (2/10)	67.5% (27/40)
% joints (n)	100.0% (40/40)	80.4% (131/163)
Tear width:		
medial % (n)		19.1% (25/131)
central % (n)	5.0% (2/40)	6.9% (9/131)
lateral % (n)		1.5% (2/131)
full width % (n)		2.3% (3/131)
medial/central % (n)		2.3% (3/131)
central/lateral % (n)		1.5% (2/131)
Tear length:		
proximal % (n)	95.0% (38/40)	66.4% (8/131)
central % (n)		3.8% (5/131)
distal % (n)	5.0% (2/40)	29.8 (39/131)
Plantar plate pathology present:		
% patients (n)	20.0% (2/10)	85.4% (35/41)
% joints (n)	5.0% (2/40)	46.6% (76/163)

^a^MRI data missing for 5th MTP joint in one patient with RA. *PP* plantar plate

High signal at the plantar plate insertion was observed in all the lesser MTP joints of control subjects; tears were only reported in two of these plantar plates. In patients with RA, high signal at the plantar plate insertion was reported in 29 of the 2nd MTP joints, 31 of 3rd MTP joints, 29 of 4th MTP joints and 23 of 5th MTP joints. High signal at the insertion was reported in 79 (60%) plantar plates without pathology, high signal at the insertion had a sensitivity of 75% (95% CI 60, 87), specificity of 9% (95% CI 4, 17), positive predictive value of 29% (95% CI 21, 39), negative predictive value 42% (95% CI 20, 66) and accuracy (percentage of exact agreement) of 31% for detecting a tear.

### Association of plantar plate pathology with disease characteristics in RA

#### Bivariable associations at the patient level

The associations at the patient level (*n* = 41) are given in Table 3. Evidence of a substantive association (Spearman’s rho ≥0.3) was reported for plantar plate pathology and Larsen score (0.60 [*p* < 0.001]) (demonstrated on MR images in Fig. 1 e, f, g and h) and plantar plate pathology and Platto forefoot structural index score (0.40 [*p* = 0.010]). Only the association between plantar plate pathology and the Larsen score would be statistically significant at the 5% level if corrected for multiplicity (threshold for significance *p* < 0.001).

**Table 3:** Spearman’s correlation coefficients patient level variables and joint level variables

	PPP (N affected/4)	Larsen score (Max)	Subluxation (N affected/4)	Callus (N affected /4)	Peak pressure (Max)
Rh factor	0.19, *p* = 0.250	0.06, *p* = 0.697	0.15, *p* = 0.363	0.03, *p* = 0.859	0.26, *p* = 0.099
RA duration (months)	0.14, *p* = 0.389	*0.42,* *p* *= 0.007*	*0.34,* *p* *= 0.030*	*0.37,* *p* *= 0.018*	*0.30,* *p* *= 0.058*
PPP (N affected/4)	-	*0.60,* *p* *< 0.001*	0.25, *p* = 0.117	0.21, *p* = 0.178	0.05, *p* = 0.774
Larsen score (Max)	-	-	-	-	0.19, *p* = 0.243
Subluxation (N affected/4)	-	-	-	-	*0.34,* *p* *= 0.032*
	Platto	FIS IF	FIS AP	VAS pain	Gait velocity
Rh factor	0.25, *p* = 0.118	0.24, *p* = 0.134	−0.10, *p* = 0.534	0.27, *p* = 0.089	0.01, *p* = 0.937
RA duration (months)	*0.56,* *p* *< 0.001*	−0.04, *p* = 0.824	0.19, *p* = 0.245	0.21, *p* = 0.183	−0.10, *p* = 0.548
Age	0.01, *p* = 0.503	0.03, *p* = 0.862	0.07, *p* = 0.646	0.08, *p* = 0.602	−0.21, *p* = 0.197
Sex	-	0.00, *p* = 0.988	0.00, *p* = 0.988	0.03, *p* = 0.829	0.02, *p* = 0.902
DAS44 (CRP)	-	*0.30,* *p* *= 0.062*	*0.47,* *p* *= 0.002*	0.02, *p* = 0.908	*−0.47,* *p* *= 0.002*
PPP (N affected/4)	*0.40,* *p* *= 0.010*	0.23, *p* = 0.152	0.22, *p* = 0.170	0.12, *p* = 0.439	−0.23, *p* = 0.157
Larsen score (Max)	*0.50,* *p* *= 0.001*	0.11, *p* = 0.508	0.18, *p* = 0.253	0.12, *p* = 0.458	−0.21, *p* = 0.194
Subluxation (N affected/4)	-	*0.50,* *p* *= 0.010*	0.26, *p* = 0.100	0.18, *p* = 0.250	*−0.33,* *p* *= 0.034*
Callus (N affected/4)	-	0.10, *p* = 0.533	0.02, *p* = 0.920	0.11, *p* = 0.483	0.11, *p* = 0.509
Peak pressure (Max)	*0.34,* *p* *= 0.029*	0.08, *p* = 0.634	−0.14, *p* = 0.389	0.18, *p* = 0.253	0.20, *p* = 0.220
Platto score	-	*0.32,* *p* *= 0.044*	0.21, *p* = 0.182	*0.31,* *p* *= 0.051*	−0.22, *p* = 0.160

*Rh* rheumatoid, *PPP* plantar plate pathology; absolute rho ≥0.3 was considered preliminary evidence of substantive association

Platto’s forefoot Structural Index incorporates deformity at the first MTP joint, which was excluded in the assessment of plantar plate pathology, and was therefore not included as a dependent variable in the multilevel modelling. In addition, clinician reported lesser MTP joint subluxation was reported as an individual dependent variable.

#### Bivariable associations at the joint level

Initial models with no explanatory variables were conducted to test for significant clustering; there was evidence of statistically significant between-patient variation in plantar plate pathology, presence of erosion, joint subluxation, plantar callus formation and peak pressure measurements.

The results of joint level bivariable multilevel modelling are given (see Additional file 1: Table A1).

In the unadjusted analyses, neither autoantibody status (rheumatoid factor) nor disease duration was significantly associated with plantar plate pathology. Longer disease duration (OR (95% CI) 1.20 (1.08–1.34), *p* = 0.001) and the presence of plantar plate pathology (52.37 (8.46–323.97), *p* < 0.001) were, however, associated with increased odds of erosion (Larsen grade > 1).

#### Multivariable multilevel associations at the joint level

The results of multivariable multilevel modelling are presented in Table 4.

**Table 4:** Results of joint-level multivariable multilevel modelling

Multivariable multilevel binary logistic regression: Odds ratio (95% CI), sig.	Dependent variable			
	Plantar plate pathology	Larsen >1	Subluxation present	Callus present
RhF positive	1.98 (0.63 to 6.23), *p* = 0.243	1.19 (0.13 to 10.56), *p* = 0.879	4.99 (0.19 to 134.46), *p* = 0.339	1.46 (0.28 to 7.71), *p* = 0.652
Disease duration (months)	1.05 (0.99 to 1.12), *p* = 0.118	1.26 (1.09 to 1.46), *p* = 0.002	1.15 (0.96 to 1.38), *p* = 0.119	1.08 (0.99 to 1.18), *p* = 0.083
Plantar plate pathology present	-	52.50 (8.38 to 326.97), *p* < 0.001	1.77 (0.45 to 6.95), *p* = 0.410	1.04 (0.34 to 3.11), *p* = 0.951
Between-patient variance	1.01 (0.27 to 3.75)	4.16 (1.12 to 15.47)	13.61 (5.03 to 36.81)	1.93 (0.49 to 7.54)
Multivariable multilevel linear regression: Percent change (95% CI), sig.	Dependent variable			
	Peak pressure (standard model)		Peak pressure (extended model)	
RhF positive	22.63 (−5.17 to 50.44), *p* = 0.111		15.16 (−12.00 to 42.31), *p* = 0.274	
Disease duration (months)	0.29 (−1.23 to 1.81), *p* = 0.709		−0.50 (−2.10 to 1.11), *p* = 0.544	
Plantar plate pathology present	−17.47 (−37.67 to 2.74), *p* = 0.090		−23.11 (−45.08 to −1.14), *p* = 0.039	
Larsen > 1	-		2.30 (−23.34 to 27.95), *p* = 0.860	
Subluxation present	-		49.48 (27.41 to 71.55), *p* < 0.001	
Variance				
Between-patient	0.05 (0.01 to 0.18), 12.5% of total		0.05 (0.02 to 0.17), 14.3% of total	
Within-patient	0.35 (0.27 to 0.45), 87.5% of tota		0.30 (0.24 to 0.39), 85.7% of total	

No substantive or statistically significant associations were found between plantar plate pathology and disease characteristics. Neither positive rheumatoid factor nor disease duration were found to be associated with the odds of plantar plate pathology, consistent with the findings of the bivariable analyses. The presence of plantar plate pathology was independently associated with an increase in the odds of erosion (OR = 52.50 [8.38–326.97], *p* < 0.001), as was longer disease duration independently associated with an increase in the odds of erosion (OR = 1.26 [1.09–1.46], *p* = 0.002).

## Discussion

This study has used an optimised high resolution 3T MRI protocol to identify plantar plate pathology in the painful forefoot of a larger cross sectional group of patients with RA, and investigate the relationship between plantar plate pathology and other features of disease. This cross sectional study has confirmed the findings from previous exploratory studies [8, 9]; namely that plantar plate pathology is more commonly reported at the 5th MTP joint in patients with RA and is associated with erosive change at the lesser MTP joints. Although the presence of plantar plate pathology was associated with an increase in the odds of erosion, the resulting large confidence intervals for the odds ratio should be acknowledged and is likely to be limited by the numbers (*n* = 41) even in this large cross sectional study. The study findings indicate that there is preliminary evidence to support an association between the consequences of inflammatory disease resulting in joint damage i.e. erosion, and mechanical changes in the forefoot (plantar plate pathology), but it would not be appropriate to draw further inferences about the strength of any association. These findings are consistent with previously reported high prevalence rates of pain and swelling at the MTP joints in early disease which then stabilize, but the prevalence and severity of forefoot joint damage increase during the course of the disease [14].

This is the first study to compare plantar plate pathology within the symptomatic forefoot of patients with RA with a group of healthy age and gender matched control subjects without forefoot pain. Although the control sample size is very small, there were significant differences between control subjects and patients with RA in the presence of plantar plate pathology at the lesser MTP joints. Tears in the plantar plate were only reported in two control subjects without forefoot pain, both identified at the 5th MTP joint in the central, distal region. Previous research has reported tears in asymptomatic non-arthritic forefeet [11] and although the aetiology of plantar plate pathology is quite unclear, speculation includes the wearing of high heels, long lesser metatarsals, hypermobility and excessive loading during sporting activities [17, 18], any of which may account for the tears seen in the control subjects. The location of tears in the lesser plantar plates in the control subjects (central, distal region) were consistent with early histological descriptions [19] in which a thin area is depicted in the central fibres at the insertion and described as a weak area or a rupture zone [20].

A tear, rupture or discontinuity of the plantar plate has been described as an increase in signal or hyperintense focus at the insertion [7, 11]. Conversely, it has been reported that increased signal intensity may be seen at the distal part of all plantar plates, immediately adjacent to the proximal phalanx insertion [21]. The presence of high signal at the plantar plate insertion on MRI in RA is not specific for a plantar plate tear, indicating that high signal seen on standard MR at the plantar plate insertion is not a useful sign [9]. Histological studies, considered to be the gold standard, would be required to confirm or refute the association of high signal at the plantar plate insertion on MRI with plantar plate pathology.

In this cross sectional study, the results of the multivariable multilevel modelling did not reveal any association between longer disease duration and plantar plate pathology. Despite improved systemic disease control, pain and deformity at the MTP joints in patients with RA remains to be a common problem [4, 5]. Recent evidence has recognised that residual disease can be present in the feet of patients deemed to be in systemic disease remission, even in early disease, putting patients at risk of ongoing damage [22, 23]. It has been proposed that inflammation of the synovium in RA can cause distension and stretching of the joint capsule which subsequently leads to instability, and with repeated hyperextension of the MTP joint during gait may predispose the plantar plates to attenuation or rupture [7]. The findings in this cross sectional study suggest plantar plate pathology is not associated with disease duration, indicating that plantar plate pathology may be associated with the residual forefoot disease reported in patients with early RA and in systemic disease remission. This further highlights the need to undertake clinical examination of the foot irrespective of the patient’s state of systemic disease remission.

In a previous MR arthrography exploratory study (*n* = 15), clinician-reported lesser MTP joint subluxation, peak plantar pressures and plantar callus formation were all shown to be associated with plantar plate pathology [9]. In this larger cross sectional study however, associations between plantar plate pathology and subluxation and plantar callus formation were not found. Reporting of subluxation at the lesser MTP joints was a subjective observation undertaken by a clinician and not confirmed with imaging techniques. The presence of callus formation was also recorded for a single moment in time and did not take into consideration recent debridement or preventative treatment that the patient may have been exposed to. Confirmation of MTP joint subluxation with imaging would be required to confirm the negative associations, and therefore the findings should be interpreted with caution.

An association between plantar plate pathology and change in peak plantar pressure at the lesser MTP joints in RA was identified. Joints with plantar plate pathology yielded mean peak pressures 23% lower than those without plantar plate pathology present (*p* = 0.039). Symptom relief resulting from off-loading the painful and deformed forefoot during gait has been identified as a characteristic feature in patients with RA [24]. The patients in this larger cross sectional study were all recruited because they complained of forefoot pain and therefore it is reasonable to assume that they may have altered their barefoot loading patterns to compensate for forefoot impairments. This may have led in turn to inadequate representation of true peak plantar pressures, despite the measurement technique itself being objective. In addition, plantar plate pathology has been shown to be more common at the 5th MTP joint and associated with erosive damage; however the 5th MTP joint is typically exposed to lower mechanical loads than the 2nd MTP joint in patients with RA [25, 26]. The predominantly lateral lesser MTP joint distribution of plantar plate pathology reported in patients with RA is consistent with the distribution of inflammatory destructive disease (erosions) at the MTP joints [12, 14], indicating that the cause is unlikely to be purely mechanical, as proposed is the case in subjects without RA [17, 18].

## Conclusions

In conclusion, this is largest current cross sectional study of plantar plate pathology in the painful forefoot of patients with RA and the first to compare the findings with healthy control subjects. The distribution of plantar plate pathology at the lesser MTP joints in patients with RA is associated with the presence of erosions in the forefoot of patients with RA and differs from that seen in healthy control subjects. The precise causative mechanism for plantar plate pathology in RA cannot be inferred from this cross sectional study; further longitudinal follow-up is required to determine the specific mechanism and presentation of pathology.

## Additional file

Additional file 1: Table A1. Results of joint-level bivariable multilevel modelling (DOCX 12 kb)

## Abbreviations

DAS: Disease activity score
DMARD: Disease modifying anti rheumatic drug
FIS: Foot Impact Scale
FOV: Field of view
IQR: Interquartile range
MRI: Magnetic resonance imaging
MTP: Metatarsophalangeal
RA: Rheumatoid arthritis
TA: Acquisition time
TE: Echo time
TR: Repetition time
TSE: Turbo spin echo
VAS: Visual analogue scale
