# Supplementary material for: Plantar plate pathology is associated with erosive disease in the painful forefoot of patients with rheumatoid arthritis
Source: BMC Musculoskelet Disord. 2017 Jul 18;18:308. doi: 10.1186/s12891-017-1668-0 (PMC5516379; doi:10.1186/s12891-017-1668-0)
Supplement: Additional file 1: Table A1. — Results of joint-level bivariable multilevel modelling (DOCX 12 kb) [file 12891_2017_1668_MOESM1_ESM.docx]

**Table A1 Results of joint-level bivariable multilevel modelling**

| **Bivariable multilevel binary logistic regression:**  **Odds ratio (95% CI), sig.** | **Dependent variable** | | | | | |
| --- | --- | --- | --- | --- | --- | --- |
|  | **Plantar plate pathology present** | | **Larsen >1** | | **Subluxation present** | **Callus present** |
| **RhF positive** | 2.04 (0.63-6.63), p=0.235 | | 1.87 (0.23-15.58), p=0.561 | | 5.94 (0.18-185.14), p=0.317 | 1.41 (0.27-7.31), p=0.684 |
| **Disease duration (months)** | 1.05 (0.99-1.12), p=0.110 | | 1.20 (1.08-1.34), p=0.001 | | 1.16 (0.97-1.40), p=0.104 | 1.08 (0.99-1.17), p=0.082 |
| **Plantar plate pathology present** | - | | 52.37 (8.46-323.97), p<0.001 | | 1.94 (0.49-7.73), p=0.349 | 1.14 (0.41-3.18), p=0.805 |
| **Bivariable multilevel linear regression:**  **Percent change (95% CI), sig.** | | **Dependent variable** | |  |  |  |
|  |  | **Peak pressure** | |  |  |  |
| **RhF positive** | | 20.76 (-6.84 to 48.37), p=0.140 | |  |  |  |
| **Disease duration (months)** | | 0.19 (-1.34 to 1.72), p=0.810 | |  |  |  |
| **Plantar plate pathology present** | | -14.30 (-32.72 to 4.13), p=0.128 | |  |  |  |
| **Larsen>1** | | -0.91 (-22.59 to 20.77), p=0.934 | |  |  |  |
| **Subluxation present** | | 42.96 (24.75 to 61.17), p<0.001 | |  |  |  |
